# Human rights and the practice of medicine

**DOI:** 10.1186/s40985-017-0054-7

**Published:** 2017-03-06

**Authors:** Dainius Pūras

**Affiliations:** 1 Centre for Child Psychiatry and Social Pediatrics, Vilnius University, Universiteto g. 3, Vilnius, 01513 Lithuania; 2 University of Essex, Colchester, England

**Keywords:** Human rights, Right to health, Mental health, Participation, Empowerment, Leadership, Patient care

## Abstract

There exists a profound disconnect and misunderstanding of the utility of human rights in the practice of medicine that demands urgent attention. The United Nations Special Rapporteur, Dainius Pūras, reflects on his career as a medical professional and why human rights in the day to day care of his patients became a powerful tool to strengthen his practice and ensure the dignity and well-being of those he served. This preface reflects on some of the troubling paradoxes encountered in the practice of medicine, identifying the disconnect between human rights and the provision of patient care as a fundamental struggle that urgently requires a framework for action, much like what is offered by the authors of this special edition. A historical reflection of the power relations between the medical profession and those it serves concludes with a message of hope and a galvanizing call for leadership from within the medical community to lead rights-based reform in patient care.

## Main text

There exists a profound disconnect and misunderstanding of the utility of human rights in the practice of medicine that demands urgent attention. In this regard, the focus of human rights in patient care in the current issue of *Public Health Reviews* is a timely and important contribution to both challenge misconceptions and present human rights as an essential tool to strengthen the practice of medicine. To my knowledge, this publication, for the first time, sets out the essential conceptual challenges for realizing human rights in patient care, uses national perspectives to highlight obstacles to the delivery of rights-based patient care, and seeks to build bridges with the health profession by boldly illustrating the impact and possibilities human rights can have on the delivery of care for some of our world’s most vulnerable groups. The scholarship and best practices compiled in this groundbreaking edition are an essential and truly practical contribution towards strengthening human rights partnerships with the health profession that can fortify the delivery of patient care that is compliant with scientific evidence and universal human rights.

My interest in human rights and also intention in 2014 to apply and serve as the United Nations Special Rapporteur on the right to health has been inspired and informed by many years of professional experience. Since 1981, I have been working as a medical doctor and university professor, with patients, their families, local and national authorities and other stakeholders, and teaching medical students and doctors. During this time, I grew increasingly concerned by a recurrent tendency in the delivery of care by these stakeholders. Specifically, I noted this tendency in the provision of care for a group of patients whom I was most interested—children and adolescents with developmental, emotional, and behavioral issues and also adults with psychosocial disabilities. Medical doctors, other healthcare providers, and decision makers too often did not consider consulting the patients regarding the provision of diagnostic and therapeutic interventions. They were driven by paternalistic attitudes, according to which they know better what is best for the patient. These doctors believed that because of these patients’ limited abilities or seeming lack of capacity, they were not able to know what is best for them. By failing to recognize the patient’s agency, care providers routinely undermined their dignity and well-being [1].

This preface briefly discusses several paradoxes in modern medicine as they relate to human rights and highlights the historical imperative for the medical profession to advance rights-based change in the delivery of healthcare.

## Human rights and the practice of medicine: some paradoxes

Healthcare systems and the practice of medicine is an area ripe with paradoxes. We live in a period of human history that has seen some of the most rapid advancements in medicine, and yet, large portions of the world’s population remain unable to access health care with even minimum standards of quality. These advances in medicine translate into health systems that, in many parts of the world, offer groundbreaking treatment for chronic diseases, yet nearly every health system fails to *deliver* such treatments to the most vulnerable and fails to sufficiently address *prevention*. Medical research in recent decades has amassed an enormous evidence base in support of non-coercive health interventions, psycho-social care, safe abortion, and harm reduction [2, 3]. Yet health laws, policies, and systems systematically deny access to these interventions and, in many countries, demand treatment protocols that contradict both science and evidence. Medical professionals are increasingly armed with comprehensive education on the practice and ethics of modern medicine, yet around the world, egregious violations of bodily integrity and human dignity are occurring at the hands of these same professionals and in the name of medicine [4].

The paradox that is perhaps the progenitor of the above examples—which I have spent countless hours pondering—is the disconnect between the provision of care in everyday medical practice and the respect for human rights principles, enshrined in the Universal Declaration of Human Rights, the International Bill of Rights, other international treaties, national laws, and constitutions.

One of the effects of this paradox is that in each country, a majority of health care professionals lack literacy of and/or enthusiasm for human rights, forcing practitioners who embrace the human rights movement to the margins. In some parts of the world, particularly countries emerging from totalitarian rule, the medical profession is deeply entrenched within the political and ideological state machinery that is allergic to the seeds of human rights. As a result, human rights lack mainstream legitimacy within the medical profession. Those wishing to utilize human rights are constrained by dual and competing loyalties and without independence to effectively integrate them into their practice.

The majority group works hard to use the tools offered by science and the practice of medicine for the care of their patients. To the most skeptical professional, human rights is empty rhetoric with no tangible benefit to the medical profession, while for others, human rights are associated with adversarial and onerous monitoring tools, which are an obstacle to their everyday work. By understanding human rights as adversarial and counterproductive, the tools offered by human rights to strengthen the practice of medicine and improve health outcomes are remain in the shadows. When members of professional medical organizations view human rights as a vessel to “revolutionize” the practice of medicine, the response is defensive, with the majority retreating to the comfort and protection of reasserting ever more vigorously those principles, long regarded as fundamental to their profession, now perceived as being under threat. This misunderstanding breeds recalcitrance, undermining the protection of human rights and impacting the health and well-being of societies across the globe (see Meier [5] for an overview of the political history leading up to the World Health Organization's invocation of human rights as a normative framework for global health governance).

## History, power, and the medical profession as a vessel for rights-based change

Time and again, care delivered under the guise of good intentions, but driven by the conviction that the duty of a health professional is to utilize all methods at their disposal to address the disease or disorder, without consideration towards a patient’s rights, will, or interests, results in troubling power asymmetries between the medical establishment and individuals in need of health care. This power imbalance precipitates patient care that is both ineffective and harmful on a *systemic* level.

Sadly, the history of medicine is not only a narrative of achievement and historic breakthroughs but also a story of expansive violations of human rights, illustrated far too often by medical practices that cause extraordinary suffering and death for countless numbers of patients [6]. Courageous medical professionals turned whistleblowers, desiring to shine a light on abusive systems, and practices have also been tragically lost to this history. It is my conviction that these systemic abuses have occurred in the name of medicine because universal human rights principles have been ignored. True change in health care systems through the implementation of rights-based approaches to health must start with a reflection upon this history and must harness the transformative potential of the health profession.

The field of mental health is an illustrative case in point. Currently, acknowledgement of the need to invest in mental health is higher on the international political agenda than ever before. This is a welcome shift in priorities given the low investment, globally, in mental health and gap in availability of mental health services [7]. However, before the international community makes bold new commitments to closing the treatment gap in mental health care, there is a vital precondition: mental health policies and services need to be liberated from their legacy of violence, stigma, and insensitivity towards human dignity [7]. Thus, the role of psychiatrists and other mental health workers is crucial to the implementation and full integration of a human rights framework in the provision of mental health care.

The care of people who experience drug dependence has been held hostage for nearly a century by prohibitionist policies shaped by outdated ideas and paternalistic attitudes towards people who use drugs [3]. This has left a powerful legacy of stigma and fear of addiction within the health profession that has negatively affected the quality and innovation of health care for drug dependence and contributed towards a global crisis in the availability of palliative care [8]. A sobering understanding of the harmful impact of drug control is beginning to emerge, with drug reform increasingly visible on the international agenda [9]. In many parts of the world, horrific and systemic abuses continue to be carried out in the name of “drug treatment” and, in many countries, at the hands of health professionals. The international medical community, availing themselves of human rights, has begun to contribute towards important progress in calls for drug reform, including advancing systemic change in how to deliver effective treatment and patient care for drug dependence [10–13]. Without health professionals as allies, there will be no sustained reform in drug treatment and access to medicines at the national levels.

## Human rights in patient care: hope for the future

The story of modern medicine is, fortunately, still being written, and there is reason to hope. Despite the fact that human rights are ignored worldwide in the delivery of patient care, the human rights movement has managed to open a new chapter that promises much hope for the practice of medicine. This chapter began 30 years ago when the AIDS movement found powerful synergies amongst human rights and health care professionals. Likewise, thanks to the mobilized efforts of national, regional, and international stakeholders, including health professionals, human rights have been at the forefront in the global reduction of maternal mortality [14–17].

These are powerful examples of how human rights fortifies the delivery of health care and how much can be achieved when the medical community is enabled to embrace human rights (even with resistance) and when all health professionals, the human rights community, and other stakeholders mobilize and make concerted efforts.

I would like to finish this preface with the words of Jonathan Mann. These words drive my activities and decisions as a Special Rapporteur and enable me to make clear decisions during my country missions and other activities. It is my greatest hope that we will see an increasing number of medical doctors and other healthcare workers in each country who are on this side:

“The human rights framework provides a more useful approach for analyzing and responding to modern public health challenges than any framework thus far available with the biomedical tradition” [18].
